# On simulation reuse in healthcare applications

**DOI:** 10.1177/00375497251383912

**Published:** 2025-11-12

**Authors:** Steffen Zschaler, Navonil Mustafee, Alison Harper, Thomas Monks, Bhakti Stephan Onggo, Christine S. M. Currie, Fiona Polack

**Affiliations:** 1 King’s College London, UK; 2 University of Exeter Business School, UK; 3 University of Exeter Medical School, UK; 4 University of Southampton, UK; 5 University of Hull, UK

**Keywords:** Simulation, healthcare, reuse, open science, model transformation, distributed simulation

## Abstract

Simulation remains a promising technology in healthcare operations research and process optimisation. However, while there have been many research projects applying simulation in this context, the level of sustained uptake in healthcare practice has been lower. We conjecture that an important reason for this is the time, cost and complexity of developing simulation models. Therefore, being able to reuse models would be key to improving uptake of simulation in healthcare. Conventional practice is that simulation models are developed *from scratch* for every new problem. In this paper, we review current strategies for model reuse in the healthcare context, aiming to identify complementary techniques for model reuse available to healthcare modellers and managers. Specifically, we identify three different modes of model reuse – forming a triadic framework – each prioritising a different aspect: FAIR and open-science aspects of model reuse; reusable conceptual simulation domains through modelling languages and transformations; and black-box model(-component) reuse including distributed simulation. We show how these three perspectives complement and enhance each other. We believe that developing concrete mechanisms and tools for leveraging the relationships between the three different modes of model reuse will be key to increasing the uptake of simulation modelling in healthcare practice.

## 1. Introduction

Computer-based modelling and simulation (M&S) is an important tool for supporting healthcare operations, strategic planning and policy analysis.^1–3^

Despite its demonstrated potential, simulation remains underused in healthcare decision-making.^2,4^ One of the long-standing challenges is the high cost, in both time and expertise, of developing new models for each application. Model reuse is the practice of reapplying existing models, components, or design knowledge.^5^ It has been proposed as a way to reduce duplication and promote uptake,^6–9^ yet in practice, reuse remains rare, largely due to issues around model compatibility, credibility, discoverability and accessibility.^6,7,9,10^ The availability of reusable simulation models in healthcare could significantly improve the use *in practice* of simulation for healthcare process improvements. Centralised healthcare systems, such as the UK National Health Service (NHS), can benefit from economies of modelling and simulation^11^ that are brought about by increasing the use of modelling and simulation in such systems through adopting model reusability principles.^4,12^

Previous research has represented simulation reuse as hierarchical, delineating various degrees of reuse ranging from complete model reuse down to minimal, function-level reuse. Robinson et al.^8^ described a simple reuse spectrum: (1) full model reuse; (2) component reuse; (3) function reuse; and (4) code scavenging, emphasising the trade-off between ease of use and complexity. Similarly, Hussain et al.^6^ propose a six-level reuse hierarchy, illustrating that while full model reuse offers maximum efficiency, it is also the most challenging to achieve due to constraints such as model compatibility and credibility. Balci et al.^10^ challenged this thinking, describing achievability increasing in the opposite direction, with reuse at the programming/class/function levels made difficult by the many options in programming languages and operating systems, while at the opposite end of the hierarchy, network-centric applications which use M&S components distributed over a network are supported by standards, such that a model built in compliance with a distributed simulation standard, such as the IEEE 1516 High-Level Architecture (HLA),^13^ can be reused by other models interconnected through the HLA protocol.

We extend this argument by rethinking earlier conceptualisations. The rise of open-source simulation libraries, modular and hybrid modelling techniques, and modern software engineering practices has changed the way models are built and shared. More broadly, the adoption of open science principles and digital research infrastructures has improved the findability, accessibility and reproducibility of simulation artefacts. These developments open up new possibilities for reuse that go beyond the scenarios described by earlier frameworks. We argue that the field now needs a conceptual update that recognises not only *what* is reused (e.g. models, code, ideas) but also *how* reuse is made possible in practice. There is an evident gap in understanding the practical methods to effectively operationalise simulation reuse, moving beyond theoretical concepts toward implementable solutions. This paper aims to address this gap by reviewing existing literature and proposing a practical, integrated framework to promote simulation reuse in healthcare contexts. We introduce a triadic framework encompassing open science approaches, modelling languages and translations, and black-box components. We show how these provide complementary answers to the challenges in simulation reuse and discuss potential synergies between these approaches. Our aim is to ignite discussion on the best practices and future directions for enhancing reusability in healthcare simulation modelling.

The remainder of this paper is structured as follows: In Section 2, we briefly summarise existing conceptualisations of simulation reuse, and review the simulation reuse research. Drawing on the insights from the literature, in Section 3, we give an overview of the proposed triadic framework that synthesises different approaches for how to reuse simulations in healthcare, before discussing their synergies in Section 4. We conclude the paper in Section 5 with some proposals for a future research roadmap.

## 2. Review of simulation reuse

Reusable simulation models or simulation model elements are often conceptualised as either generic or generalisable. Boyle et al.^14^ differentiated between these two types, defining generic models as those developed from theoretical principles without reliance on specific empirical data,^15^ intended primarily for conceptual exploration and general problem-solving. In contrast, generalisable models can be adapted using site-specific data, allowing for broader applicability and more practical reuse across different contexts. This flexibility aims to balance the initial development effort with subsequent reuse benefits.

While this differentiation between generic and generalisable models offers a useful way to characterise what may be reused, it does not fully explain how such reuse occurs in practice or what conditions support it. To better understand the current landscape of reuse-enabling practices and tools, we conducted a search of the recent simulation literature to identify empirical examples of model reuse. We conducted a structured literature search using Web of Science (search terms: ‘model reuse’ AND (DES OR ‘discrete event’ OR ‘discrete-event’ OR ABM OR ‘agent-based’ OR ABS OR ‘agent based’)), which returned 43 results. Following full paper review, 18 papers were excluded due to lack of relevance to simulation reuse, being outside of applied contexts, or using simulation methods that are outside of Operational Research, such as CAD or 3D geometric modelling. Of the 25 remaining papers, we examined:

- Techniques for simulation/model reuse;
- The mode of simulation reuse described;
- Challenges for simulation reuse;
- Specific application domains.

The review identified a variety of reuse strategies that go beyond standard typologies. These include generalisable model frameworks, experimental reuse of pre-built models and modular cognitive architectures. Two studies proposed distributed simulation frameworks that support model reuse by enabling federation of existing models.^16,17^ Several studies emphasised the role of modelling languages and translations in enabling reuse. These include XML-based description files for structured model generation,^18^ model transformation between DEVS and SMP2 for interoperability,^19^ and translation across formalisms (e.g. SDL to DEVS) for cross-verification.^20^ Others propose reusable frameworks for PDES,^21^ abstract simulators^22^ and executable enterprise architectures using standardised representations.^23^ Together, these show the growing emphasis on formalism-bridging, modularisation and abstraction as enablers of simulation reuse.

Two healthcare-focused studies support the role of open science in enabling reuse. Penn et al.^24^ described the reuse and redevelopment of a generic hospital ward model, highlighting trade-offs between flexibility and simplicity. Harper et al.^25^ explored how simulation models developed in Python could be made more accessible to non-technical users through deployment strategies. Both studies identified barriers such as complexity, lack of standardisation and limited accessibility, reinforcing the need for reusable models to be openly shared, documented and usable without specialist skills.

Across diverse domains, several studies offered practical insights into simulation reuse, for example Monks et al.^26^ experimentally explored trade-offs between model reuse and client learning, while Petersen et al.^27^ introduced modular components for biomedical simulations. Technical strategies include component-based modelling,^28^ formal pattern reuse via Petri Nets,^29^ and interoperable DEVS components.^30^ Some of the challenges of reuse of agent simulations of complex immunological behaviours, were examined with a demonstrably fit-for-purpose scientific simulation.^31,32^ Other work focused on cognitive architecture standardisation,^33^ integrated modelling for nuclear policy,^34^ or hybrid frameworks that blend simulation with contextual organisational insights.^9^ Challenges such as model familiarity, validation, documentation and stakeholder trust recurred frequently.^35,36^

Collectively, these studies highlight reuse as technically feasible but often limited by practical, organisational and epistemological barriers. Despite an established conceptual understanding of reuse, practical mechanisms for operationalising simulation reuse remain underdeveloped. There is a clear need for methodologies that effectively address these identified challenges, providing frameworks that support not only theoretical conceptualisation but also practical implementation. Drawing on these insights, we propose a triadic framework that synthesises open science principles, modelling languages and black-box components methods. These have been identified across the literature as recurring enablers and complementary strategies to support practical and effective simulation reuse in healthcare.

## 3. Three different approaches to simulation reuse

In this section, we briefly describe three specific approaches to reusing simulation models. In the next section, we will discuss how these approaches inter-relate.

### 3.1. Open science tools to enhance the reusability of computer simulation models

The general aim of open science is to make research widely accessible, promoting the transparency, credibility and reuse of computational artefacts.^37,38^ In healthcare, code sharing and other open practices are seen as a way of providing a transparent, unambiguous record of analysis,^39^ but also maximising limited resources and increasing the potential for impact.^40,41^ Barriers to sharing healthcare simulation models stem primarily from time, skills and infrastructure required to prepare code for reuse.^42^ Broader surveys of healthcare research practice echo this, indicating that although researchers are willing to share code, actual reuse is constrained by documentation quality, lack of persistent repositories and limited incentives.^43,44^ While code reuse is most frequent among colleagues and within trusted networks, wider adoption is inhibited by these structural challenges. It follows that open science practice enables model reuse in healthcare.^45^ In this context, we discuss four sets of resources available to the simulation modeller to enhance the open science credentials and reusability of their models. These approaches are mainly, but not exclusively, applicable to models built using free and open-source software (FOSS) such as Python or R.

#### 3.1.1. Discoverability and preservation

Making a model available, and – as importantly – discoverable, makes it more likely to be used in new applications. Across healthcare organisations, we see similar processes, problems and data structures.^9,14^ Model reuse helps to raise the profile of the work and increase both its academic and practical impact. A contemporary way to do this is archiving through a digital open science repository. This approach also preserves the model, as archives such as Figshare, Zenodo and the Open Science Framework provide guarantees on persistence. Benefits for modellers include citable models using a Digital Object Identifier (DOI),^46^ and meta-data that increases the likelihood that a model is discovered.^40^

#### 3.1.2. Frameworks

Guidance, software and templates exist to support the structuring of simulation models and accompanying artefacts to enable open modelling in healthcare. For healthcare discrete-event simulation modelling, STARS (Sharing Tools and Artefacts for Reusable Simulations)^45^ aims to overcome barriers to sharing models in healthcare by providing essential (such as open licensing) and optional (such as enhanced documentation) components to strengthen the reusability of computer simulation models written in free and open source software. The authors provide three applied simulation examples in Python. In an area related to simulation, Smith et al.^47^ provide packaging guidance for Health Technology Assessment models in R. More generally, van Lissa et al.^48^ developed WORCS (Workflow for Open Reproducible Code in Science), a structured framework to support an open science workflow in R. Each of these support the necessary activities required to develop models that are findable, available, executable, modifiable and reusable.

#### 3.1.3. Model reporting guidelines

Reusing a simulation model will often include some adaptation of the current version to allow for differences in the system, making clear documentation vital. Various guidelines exist for describing simulation models such as STRESS (Strengthening the Reporting of Empirical Simulation Studies)^49^ which has checklists for discrete event simulation, agent based simulation and system dynamics; the popular ODD protocol^50^ designed specifically for agent based simulation; and RAT-RS^51^ which focuses on documenting how data is used in agent based simulation. Reporting guidelines document the essential steps, inputs and outputs of an M&S lifecycle, supporting reproducibility of results, interpretability of the model, and model reuse. They ensure that a manuscript can be understood, and a model and its results can be replicated and trusted. In healthcare research more widely, this is seen as a priority; for example, the Equator Network^52^ is a repository of healthcare research reporting guidelines, aiming to improve the reliability and value of published health research literature through transparent and accurate reporting. The US Food and Drug Administration (FDA) and other medical certification authorities are moving towards defining clear standards for simulation validation and documentation.^53,54^

#### 3.1.4. Evaluation

To confirm if a model is reusable, an option for authors is to submit the computer model for review. Such a review may award badges; for example, ACM journals operate a replicating computational results initiative, where a peer-reviewer will assess the reusability of the model, write a report, and then has the option to award an ACM RCR badge: ‘Artefacts Evaluated – Reusable’. This provides confidence to potential re-users that the model is in a state where it could be adopted for their project. Models are only validated for their intended purposes. Other application domains such as defence have explored accreditation of models by an independent third party,^55^ but to our knowledge, no such scheme exists within healthcare.

#### 3.1.5. Open science examples

While not specific to simulation, HDR UK^40^ provide a set of development principles and a GitHub repository of open-source code in healthcare research. They aim to work with academic partners to enable reuse in healthcare, where new development is seen as an exception, rather than a default. With a focus on discrete-event simulation, Monks et al.^45^ provide several example applications implementing their STARS framework in healthcare. A tutorial demonstration model^56^ implements an urgent care treatment centre simulation using all of the STARS optional components including enhanced documentation that is hosted online, and a web app to support usability by NHS users. An applied example of a generalisable, reusable model with a web app interface was developed for orthopaedic resource planning,^57,58^ and for operating theatre efficiency,^59^ both for NHS users. While still in the minority in M&S, more examples of M&S open science are starting to be seen in the academic literature.^42^

### 3.2. Reusability using modelling languages and translations

Various authors have proposed enabling simulation-model reuse by encoding knowledge about a particular domain in domain-specific modelling languages (DSMLs)^32^ or by extending existing general modelling languages (e.g. BPMN)^60,61^ from which simulations can be automatically generated,^32,62–64^ or where simulations can interpret the models as they run.^65,66^ Together, the DSML, extended general modelling language (EGML) and the program for generating the actual simulation encode a reusable simulation domain – new simulation models can easily be created by capturing their specifics in the DSML or EGML, and then generating a simulation. In other words, a simulation model can be represented using a modelling language (DSML or EGML) which is independent of the software that runs the simulation model. One main benefit of this approach is that we do not have to re-code the simulation model every time we need to change the simulation software. Thus, it will help the healthcare organisations to avoid vendor lock-in. Consequently, the DSML or EGML representation of the model can be viewed as an organisation long-term asset. Models captured in this way enable a form of *grey-box reuse*: we do not reuse the full model ‘off the shelf’, but instead there is a way for explicitly capturing what is specific about the present healthcare setting, while aspects that remain the same across settings can be reused as part of the modelling language and the simulation generators.

Below, we will discuss two approaches: (1) by extending an existing general modelling language, and (2) by creating a DSML.

#### 3.2.1. Reusability using EGML: BPMN and UML

Several authors propose to extend existing general modelling languages for various applications including for healthcare.^60,61^ Some general modelling languages such as the Unified Modelling Language (UML; https://www.uml.org/) and Business Process Model and Notation (BPMN; https://www.bpmn.org/) have been widely used in industry and provide specification and conformance standards. Therefore, it is not surprising that authors have proposed to extend UML and BPMN to support applications in healthcare.^63,67,68^

Although process modelling has increasingly been integrated into healthcare management process, BPMN and UML have not been widely used in healthcare applications,^61,69^ especially for executable simulation models. This may be attributed to healthcare being a highly regulated sector as well as the complexity of healthcare processes. Pufahl et al.^61^ identified nine challenges when modelling complex healthcare processes: BPMN cannot be used directly to model some of the complex healthcare processes. To address this, authors have proposed various BPMN extensions that can be generalised into three groups: patient-related extensions, medical practice-related extensions and resource-related extensions.^61^ Until these challenges are addressed, the work on executable simulation models will be limited to modelling simpler processes. Software tools such as Bizagi, Visual Paradigm and Simul8 can simulate simple models represented in BPMN with the help of standards such as BPSim (bpsim.org). Some examples of BPMN simulation in healthcare include emergency department^70^ and orthopaedic outpatient clinic, surgery and neurological theatres, ageing and complex medicine (inpatients).^62^

Research into making UML executable is more mature than BPMN^71^ partly because UML was invented earlier than BPMN. The latest UML specification was followed by the introduction of the Foundational UML Subset (fUML; https://www.omg.org/spec/FUML) and Action Language for Foundational UML (AlfUML; https://www.omg.org/spec/ALF) have enabled us to make a model represented using UML executable. Hence, it is possible to represent a simulation model using UML with fUML or AlfUML. However, there is little research that applies this to simulation in healthcare. Some work is using UML to represent healthcare models but only for communication purpose; the translation to software is manual. Examples include fractured neck of femur care process^72^ and Emergency Department.^73^

The above examples show the benefits of using an existing general modelling language that has become a standard to enable simulation model reuse in healthcare. Using a standard language means that the model represented using the language will be understood by stakeholders from various backgrounds who are familiar with the standard. If some stakeholders are not familiar with the standard, it is arguably easier and cheaper to provide training over a period of time for a standard than for multiple different simulation software systems. As more people in an organisation learn the same standard modelling language, knowledge accumulates over time, making it easier to share knowledge within the organisation. This is especially true for a healthcare organisation whose main business is not developing simulation models. Furthermore, a widely used standard modelling language (e.g. UML and BPMN) has good resources, such as tutorials, documentation and discussion fora, available for public access. Further benefits include that any software tool that conforms to the standard will provide the same notations for the same simulation components, making communication and training on the simulation software easier; and that a standard typically has a longer shelf-life than a specific software simulation. Note that simulation software is typically updated repeatedly, and at some point backward compatibility is lost, forcing organisations to deal with legacy models that can no longer be run; using a standard modelling language however, new executables can be generated by providing a new or updated translator (or model transformation) to the required simulation software version. Hence, a model written in a standard modelling language can become a long-term asset for an organisation.

The main disadvantage of using an existing standard modelling language is that it is not specifically designed for simulation modelling. Therefore, the standard needs to be extended before it can be used for simulation modelling. Zarour et al.^60^ provide a comprehensive review on how BPMN has been extended in various domains including healthcare. Another alternative is to develop a domain specific modelling language (DSML).

#### 3.2.2. Reusability using DSML: ED modelling language

Godfrey et al.^74^ describe a DSML particularly catering for the simulation of workflows in emergency departments. Their language is based on notations observed to be used by ED staff to make decisions about alternative workflows and communicate agreed workflows to all members of the ED – specifically, PowerPoint slides with a flow-chart–like notation called an Action Card. The language provided by Godfrey et al.^74^ closely mimics the structure and visual notation of these action cards and provides additional concepts to capture information about patients, tests used and their properties, physical department layout, etc. From models expressed in this DSML, a simulation implementation in Repast is automatically generated with the ability to address questions such as the balance between cost, patient risk and length of stay for different action cards.

Godfrey et al.^74^ report that developing new simulations in the ED space was quite straightforward once the domain specific language was created and that it was easy to adapt models for new contexts – as long as the new context could be captured within the vocabulary provided by the DSML. More generally, a DSML may not cover all aspects of a new context, but it is straightforward to extend the coverage of the DSML, such that significant parts of the existing models can be reused.^75^

DSMLs are not limited to capturing the vocabulary of the healthcare domain. Zschaler and Polack^32^ illustrate other aspects of simulation models that can be expressed – for example, arguments of validity (fitness for purpose), or descriptions of the simulation experiments to be performed. This makes these aspects amenable to formal scrutiny and (semi-)automated execution and evaluation.

Validation is a key consideration in simulation reuse. If validity arguments are explicitly modelled (e.g. using a form of structured argument),^32,76^ it becomes possible, in principle, to explicitly trace the impact of changes to the model to parts of the validity argument that need to be reconsidered or that may be reused – though the details of this remain to be studied in depth.

Reproducible execution of experiments is essential for simulation reuse. DSMLs for the description and automated execution of simulation experiments^77,78^ make it easier for experiments to be performed consistently irrespective of who runs the experiment. Once an experiment-specification language has been established, it becomes possible to consider generating simulation experiments^79^ from queries formulated in a suitable query-modelling language.^32,80^

The DSML approach differs from the previously presented ones in that it uses the language of the domain (e.g. vocabulary familiar to ED staff) rather than the generic concepts of the modelling language (UML, BPMN). This has clear benefits in that clinical staff are directly able to interact with the models without requiring training in BPMN or similar standard notations, which are typically not used directly by clinical staff. The DSML is also directly matched to the needs of the healthcare domain and does not need further adaptation as is the case when using general modelling languages. As such, using a DSML can increase reusability of a simulation model as it can be maintained and adapted to changes in the real-world processes directly by clinical staff. Note a DSML is often created on top of a standard modelling language, giving access to the tooling and practices where appropriate. This means, for instance, that existing BPMN models of relevant processes can be imported into a DSML model.

### 3.3. Reusing simulation models as black-box components

Black box reuse is common in software engineering,^81^ and we have also found several papers in the literature that employ black-box reuse in simulation modelling.^16,17,26,28–30^ These approaches typically treat entire simulation models as a component, which is reused without inspection or adaptation of its implementation details. Black-box model reuse often involves the composition of multiple model components.

The multiple model components may be owned by different departments or organisations. Therefore, more often than not, the model components are distributed over a network. Since the 1970s, the Parallel and Distributed Simulation (PADS) community has developed novel approaches to *distributed simulation*, which is the distributed execution of simulation over multiple computers linked over a geographical network, and *parallel simulation*, the use of parallel computing for faster execution of a single simulation over multiple processors.^82,83^ These distributed simulation standards, techniques and best practices, thus, offer excellent tools for black-box reuse and linking of healthcare models. However, specialised software must exist that coordinates the execution of the simulation time between existing models.^82^ In PADS terminology, the software is referred to as distributed simulation middleware. The IEEE 1516 Standard for M&S High-Level Architecture (HLA), which has become the de-facto standard for distributed simulation, refers to individual simulation systems as federates, which, together with the Run-time Infrastructure (RTI, the middleware that enables message exchange and simulation time synchronisation among federates) and the Federated Object Model (the model defines object and interaction classes), form an HLA federation.^13^ The IEEE 1516 standard has been used for example to implement a blood supply chain network consisting of one central blood processing, testing and issuing (PTI) centre and multiple hospitals, with each sub-model developed in a commercially available simulation package (CSP) – Simul8 – and implemented as an individual HLA federate, which together, through use of the HLA-RTI 1.5NG middleware, act as a distributed simulation federation.^84^ The study by Anagnostou and Taylor^16^ developed a distributed simulation of an emergency medical service based in London. The model was a hybrid agent-based and discrete-event simulation; the HLA RTI was the Portico v2.0 RTI implementation of IEEE 1516-2000.^16^

Healthcare simulation models have traditionally been developed using CSPs like Simul8, Flexim and Arena. Implementation of a distributed simulation using CSPs that do not readily support distributed execution of models can be a challenge.^84^ Thus, the question is why distributed simulation is considered a feasible approach for model reusability. To answer this, we first refer to some general literature on cost and time for model development, model validation, and so on, and then argue the opportunities that distributed simulation presents in healthcare M&S.

First, following the life cycle of a simulation study, we can identify various cost elements that a project is likely to incur. For example, an M&S expert/consultant may need to be hired; in the conceptual modelling phase, the stakeholders’ time has a cost element; model implementation may require the purchase of commercial off-the-shelf software or support; there are likely to be costs towards collecting data, especially if this is primary data. Large and complex models may need several years to reach optimal point in terms of artefact development and would have benefited from several rounds of verification and validation, and step-wise introduction of organisational knowledge, all of which enhances different aspects of trust between the stakeholder, modeller and the model.^85^ Distributed simulation enables the reuse of such black-box models; these are the trusted artefacts, developed over several years in individual healthcare organisations, and the likelihood of trust being eroded due to the model being a part of a larger distributed model is low. However, it is acknowledged that the distributed simulation as a whole (simulation federation), which is composed of sub-models, will need additional interventions for the broader group of stakeholders who are now associated with the larger modelling initiative; this is especially the case for modelling artefacts that are from other organisations. To elaborate on this, we take the example of neonatal treatment units (NTUs) in hospitals that are served by one human milk bank (HMB); the former models the processes around ordering and inventory management of perishable milk stocks in NTUs, while the HMB models splitting strategies for the batches of donated milk and allocation of stocks to NTUs.^86^ A distributed simulation model of such a milk banking network can thus consist of several NTU models that are trusted by the hospitals and linked to the HMB model. However, for NTUs’ stakeholders to trust the HMB model, and vice versa, may take additional time and efforts.

**Figure 1.**
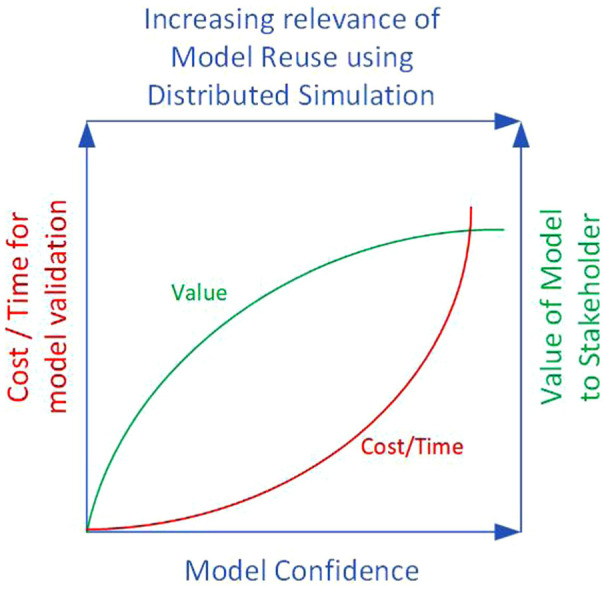
Increase of stakeholder trust in models, frequently brought about through the investment of money and time towards model validation – adapted from Sargent^87^– increases the opportunity of reusing trusted models using distributed simulation approaches.

Next, we frame the utility of the distributed simulation approach of whole model (black-box) reuse with reference to the literature on model validation. Sargent^87^ discussed the relationship between model confidence and the cost of validation, the amount of time needed for validation and, finally, the value the stakeholder would derive from the model. To clarify this relationship the author used a figure that comprised three axes: cost/time, model confidence and value of the model to the stakeholder. It illustrated that higher model confidence would incur higher costs towards model validation, but the marginal gain of value to costs would be steadily increasing; however, after a certain threshold of confidence is reached, the marginal gain in stakeholders’ value attribution of the model compared to validation costs will experience a steady decline.^87^ Thus, it is arguable that for a model that has incurred significant investments in time and costs towards model validation, whole-model reuse using distributed simulation standards and middleware presents an additional layer of opportunity. This is shown by extending Sargent’s conceptualisation, adding an additional axis to depict the increasing relevance of model reuse using distributed simulation (Figure 1). We take the intersection point between value and cost/time curve as an example. At this point, the value of the model to the stakeholders is high since there has been an investment of both time and costs towards model validation, which has increased model confidence. The relevance of distributed simulation for model reuse is also high at this point.

We take the example of healthcare supply chain simulation to discuss opportunities for DES model reuse. A healthcare supply chain consists of several tiers, such as manufacturers of pharmaceutical products, Tier-1 suppliers such as Active Pharmaceutical Ingredients (API) suppliers, drug distributors with warehouse and logistics functions, and retailers like hospital pharmacies and community pharmacies. A drug manufacturer *(M)* may routinely employ a DES model for planning the production of pharmaceutical products; similarly, a distributor *(D)* may use a DES model to simulate the flow of various drugs through its network of warehouses. *M* may be considering the services of a new distributor through a tendering process. *D* may be a contender for the tender. Since both *M* and *D* employ DES models for the planning of their day-to-day operations, the use of simulation in the tendering process to model the future pharmaceutical production planning, inventory and distribution functions can be considered. This may allow opportunities to experiment with supply chain shocks and determine the resilience of the existing systems, and how the processes in the two organisations may react if they are joined through a successful tendering outcome.

A DES distributed simulation federation composed of the individual DES models in use at *M* and *D*, respectively, could enable experimentation of such future *M–D* supply chain operations. Reusing such healthcare DES models through distributed simulation would arguably be the preferred option compared to developing a new supply chain simulation that integrates the processes for both M and D. The distributed simulation approach treats each model as a black box; when compared to conventional supply chain simulation, its distributed counterpart enables model reuse, data privacy/information hiding, data integrity and opportunities for speeding up the execution of large models.^88^ A new model will also take time and money for model validation and to gain the trust of the stakeholders.^87^ Finally, distributed simulation enables model reuse, and thus, trusted artefacts are used.

## 4. Discussion: the triad of reusability

Existing studies on model reuse^6,8^ have focused on the costs and benefits of reuse in relation to *what* part of a model is being reused, ranging from code scavenging (reusing individual pieces of model code in a copy-and-paste fashion) to full model reuse.

**Figure 2.**
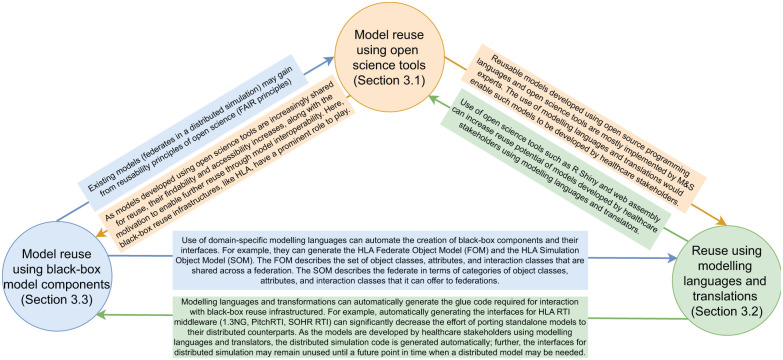
The triadic relationship between three modes of simulation re-use.

In our choice of types of model reuse, we have focused on *how* models or model components are being reused. The spectrum here ranges from white-box reuse in the spirit of FOSS to black-box reuse of model components, with an intermediary form of grey-box reuse with modelling languages and transformations. We also found some examples of generic models – models that aim to cover many possible healthcare contexts with minimal parametrisation need. These approaches conceptually sit between grey-box reuse with modelling languages and transformations, and black-box reuse with model components: whether they are closer to one or the other depends on the complexity of the parametrisation available (simple numeric parameters in black-box reuse and potentially complex model structures in grey-box reuse). This leads to a triadic model of model reuse *modes*, which are summarised in Figure 2:

1. The first element of our triad of model reuse focuses on *model reuse using open science methods*. The popularity of FOSS packages for simulation like SimPy, Salabim, AgentPy and Mesa (all in Python) has also meant that the current generation of modellers is more code savvy. This has meant that developers of models and simulations have become less dependent on CSPs. Although CSPs continue to flourish, there is an increasing recognition that an extension of the current state-of-the-art in M&S, through, for example, the development of hybrid simulation models^89^ that integrate approaches such as using discrete-event simulation (DES) and machine learning, necessitates a certain degree of flexibility in the use of software. This flexibility is more readily provided using FOSS software and libraries. Developing complex DES models using CSPs requires expertise in either the CSP-specific programming language – for example, Visual Logic for Simul8 – or a general-purpose programming language (e.g. C++ for FlexSim). However, such coding generally tends to be accomplished within the parameters of the overarching CSP, to the extent that interrogating the next event in the event list (an event list is a data structure that consists of the time-stamped ordering of future events which is maintained by a core element of the simulation software which is referred to as the simulation executive or the simulation engine) from an external program could not generally be accomplished without vendor support. In contrast, FOSS DES software and libraries provide access to simulation executives through pre-classes and function calls, which could be more readily integrated into the code base. Thus, with the advent of FOSS and its increasing use in both academia and practice, we recognise that the simulation modeller of today is arguably more adept at developing complete simulations in, for example, Python and R with DES or ABS libraries, which, we argue, significantly extends the piecemeal use of coding that is necessary when introducing complex logic in standard models developed using CSPs. Thus, full-model reuse, as opposed to code scavenging, may no longer be the most challenging approach, different from the observations of Robinson et al.^8^
2. The second element of our triad of model reusability is *black-box reuse of model components*. Component-based simulation is a modular approach to building computer models, where individual components are developed separately, with the intention of model reuse, and interact through well-defined interfaces. When the internal workings of these components are either unknown or irrelevant to the simulation study, this approach is known as black-box component model reuse. A common example is found in simulation federations, where multiple self-contained sub-models operate together within a larger system. In such cases, distributed simulation enables black-box integration by managing data exchange between components through exposed interfaces, without requiring access to their internal logic.

In the literature, we find examples of standards on the use of distributed simulation methods and approaches with CSPs, in particular, the *SISO CSPI PDG Standard for COTS Simulation Package Interoperability Reference Models*^90,91^ and the use of the IEEE 1516 HLA standard.^13^ However, the use of distributed simulation in the industry has only had limited success. Our discussion on CSPs in relation to FOSS and open science is relevant here. Integration of CSPs with external applications may require bridging software for message passing. For example, Mustafee and Taylor^92^ grid-enabled CSP Witness with their *WinGrid* desktop grid computing middleware through the use of software adapters that relayed messages (and callbacks). Similarly, developing a distributed simulation with CSPs usually necessitates the development of ancillary artefacts that couple the CSP implementation of a DES model with the distributed simulation middleware. A middleware based on a standard such as the IEEE 1516 will have open interfaces for communication which are well documented. However, CSPs that are generally used to develop healthcare models are not architecturally designed to support distributed simulation or other forms of black-box model reuse; further, relevant documentation and APIs may not exist that readily allow such reuse. For example, the ancillary software that serves as a bridge between the CSP and the distributed simulation middleware needs to be manually coded. The success of distributed simulation in space and defence, for example, could be partly attributed to the programming of the simulation model (rather than the use of CSPs), making the integration with the distributed simulation software easier. This is not yet prevalent in healthcare. The use of FOSS presents us this opportunity!

3. The final element of our triad supports *model reuse through modelling languages and translations*. Here, we need to differentiate the roles of *software developers* (programmers, CSP vendors and researchers), *model developers* (M&S experts, who may be practitioners or researchers) and *model users* (stakeholders). Importantly, MLTs enable healthcare stakeholders to be directly involved in model building and parametrisation. To enable this, the developers of the MLTs may organise workshops and guided learning sessions for stakeholders, who then learn the modelling language and gain the skill of developing models based on taxonomies they associate with (which is again dependent on domain) rather than M&S constructs. This is typically not possible if either CSPs or FOSS tools are used for model development. For more complex problems, stakeholders and researchers can co-develop the models in a participatory modelling setting.^62^ MLTs enable better communication between stakeholders and researchers which is critical for model co-development or participatory modelling. Stakeholders may be able to input parameters for models developed using CSP or FOSS if, for example, the frontend provides relevant functionality. Importantly, MLTs shift implementation effort to the core software artefacts, which need to be developed for particular healthcare domains. This is similar to the skill set in programming necessary for the deployment of distributed simulation techniques and FOSS for furthering the reuse of simulation models.

Table 1 provides a high-level comparison of the three reuse modes according to several categories. The first three columns focus on different stages of model development and reuse, capturing who undertakes the work and what they are developing. Here, it is noticeable that black-box reuse is primarily the work of M&S experts, including the parametrisation stage. In contrast, open-science-based model reuse, and even more so MLT-based reuse allows a shift away from M&S experts and towards healthcare stakeholders for model building and parametrisation. The key differentiator here is the complexity of what healthcare stakeholders can contribute directly: in MLT, they can potentially contribute full models, whereas in open-science based reuse this quickly requires a degree of programming expertise (e.g. available to data scientists/analysts). Another differentiator between open-science-tool based reuse and MLT-based reuse is that well-designed domain-specific modelling tools (in the MLT case) provide a degree of integrated self-documentation, whereas open-science tools often require substantial separate documentation to be made available.

**Table 1.** Properties of the three reuse modes.

Reuse mode	Core software artefact (what and who)	Model building(what and who)	Model parametrisation(what and who)	Applicable studysettings	Real-world uptake	Barriers for uptake	Tool support	IPR considerations
**Open Science Tools**	Typically a model built by M&S experts using FOSS with accompanying documentation	Data scientists/analysts: direct adaptations to the model code; Other healthcare(re-)users: parametrisation through a web-based ‘dashboard’ interface	Details of model parameterisation should be provided as part of the documentation to enable reproduction of experiments	All study settings. Where data are confidential then synthetic data may be provided for testing.	Growing uptake in healthcare, examples include reusable models with web-browser interface, and documented open models with dependencies locked down	Confidentiality constraints, lack of incentives to share code, limited technical capacity among stakeholders, and usability challenges for non-technical users	Frameworks (e.g. STARS), infrastructure (e.g. GitHub, Zenodo, Docker) and tools (e.g. R Shiny, Streamlit, WebAssembly)	Requires clear licensing and open-access documentation. Institutional IP policies and lack of licensing clarity may restrict reuse
**Black-box model components**	M&S expert developers of model components	M&S experts by composing existing models	Mostly M&S experts by providing parameters to model components	Where internal model structure is confidential	Limited in healthcare, but examples in domains such as space and defence	The use of black-box approaches, such as distributed simulation, often requires expertise in applied computing and software engineering; this extends beyond the traditional OR skill-set needed to develop the simulation model	The integration of black-box approaches with commercial simulation products is an area of research. Arguably, commercial vendors have not introduced proprietary tool-level support due to a lack of demand and vendor lock-in	Standards such as IEEE 1516 HLA exist. The implementation of the standards can be protected by copyright, even though the standard itself is public
**Modelling Languages and Translations**	Domain-specific modelling tool developed by M&S expert	By healthcare stakeholder (using modelling knowledge encoded in domain-specific modelling tool or using participatory modelling with M&S expert)	Healthcare stakeholder – support for parameters more complex than single values; Domain-specific modelling tools are partly self-documenting	Settings with well-established understanding of a general and recurring model structure; participatory modelling settings with stakeholders and M&S expert	Limited in healthcare, but examples in other domains (standard in engineering, but also emerging in airplane fleet management and supply-chain management), BPMN extensions for healthcare, DSML for healthcare	Initial cost of developing domain-specific modelling tool, Higher skill level required for tool creation, cost to maintain translation software	E.g., Eclipse Modelling Framework and related ecosystem, Bizagi, Visual Paradigm, Simul8	Vendor lock-in through reliance on domain-specific modelling tools, which may impose proprietary file formats

The fourth column briefly discusses characteristics of study settings that make the different reuse modes more or less applicable. Part of the differentiation here is related to confidentiality of model structure – a topic that is further discussed in the final column on IPR considerations. Here, naturally, black-box reuse enables tighter protections of confidentiality and IP than open-science tools would. However, generation of synthetic data can go some way to addressing this issue. MLT-based reuse requires a significant degree of understanding of the study setting, ideally in situations where the likelihood that models will be reused in different contexts is high. As mentioned before, MLT-based reuse also naturally enables participatory approaches to model development.

The next three columns describe issues related to real-world uptake. We give some brief notes on the real-world uptake of each reuse mode and list some tools available to those wishing to create models based on a particular reuse mode. We also describe some of the key barriers to uptake. For open-science tools, these include issues of confidentiality, as noted above, but also a systemic lack of incentives to share code openly. Open-science reuse, being white-box, requires significant programming ability of reusing stakeholders. This is also often true of black-box reuse approaches, not least distributed simulation, which requires the use of complicated technical infrastructures for reuse. This is not always available and this is where MLT-based reuse comes in by providing ways of capturing models closer to the stakeholders’ expertise. However, MLT-based reuse comes with a higher initial cost for model development, as there is a need to develop the initial domain-specific modelling tools and not just an individual model.^75^

Finally, we briefly discuss IPR considerations. Clear management of licensing is critical to all three reuse modes, but most importantly in the context of open-science-based reuse, where open-access documentation is also central. Issues with IP management can limit the ability to reuse individual models. For black-box reuse, this is somewhat mitigated in that the individual component models are more easily protected. Even specific implementations of the reuse infrastructure can be copyright protected, even though the relevant standard is public. MLT-based reuse methods, like black-box reuse, can create a degree of vendor lock-in, as they depend on specific technologies and tools (possibly including proprietary file formats) for model development and reuse.

### 4.1. Hybrid reuse modes

The three modes of model reuse are complementary. Next, we discuss the synergy opportunities created by combining two modes of reuse (cf. Figure 2).

A synergy for model reuse can be identified between black-box model components and FOSS, especially since the development of models in both approaches requires programming knowledge and, thereby, accords the programmer the flexibility of incorporating some of the Findability, Accessibility, Interoperability, and Reusability (FAIR) principles of Open Science tools and making distributed models findable and accessible.^93^ This could be achieved, for example, by making distributed simulation federates (each a complete simulation model) available and documented through code repositories and open science archives.

Open Science methods for DES model development can similarly benefit from the interoperability and reusability afforded by black-box model component infrastructures. For example, as model reuse becomes more widespread and complex models start to be routinely developed using FOSS tools, there is likely to be the motivation to use distributed simulation techniques to weave complex simulation federations consisting of available and accessible sub-models, an extension of model reuse which is readily achieved using FOSS programming languages and open science tools at one end, and implementation of distributed simulation standards and APIs at the other.

Next, we consider how MLTs and FOSS tools leverage reusability. A unique aspect of MLTs is the stakeholder taking the role of model builder (Table 1). As modelling languages are domain-dependent, we can consider (say) a modelling language for healthcare developed for a specific regional authority; however, it is arguable that the language will also be largely understood by professionals working in the same domain and across geographies. This increases the potential of model reuse manifold through FOSS implementation of MLTs and the underlying (target) simulators, making them available through open science FAIR principles and related technologies – for example, packages to develop model interfaces, web deployment and web assemblies. Another benefit of this combination is that those who plan to reuse the MLTs do not need to be restricted by the target simulators.

Conversely, MLTs can easily generate the artefacts required to support FAIR principles, making it easier to package simulation models ready to be reused.^94,95^

Finally, with respect to furthering model reusability by combining the approaches on black-box reuse and MLTs, the use of domain-specific MLTs offers the opportunity for automation the generation of interface specifications,^96^ such as, for example, the HLA Federate Object Model and the Simulation Object Model. For UML-based MLTs, open source tools such as Papyrus provide an execution environment that can communicate with HLA-compatible applications.^97,98^ On the other hand, the developers of MLTs can implement translators using the principle ‘distributed simulation artefacts by design’, whereby the software generates the interface code for time advancement and message passing; the latter is implemented when such MLT-designed models may need to be linked, making the model reusable as a black box in the HLA model component infrastructure.

Most of the triadic relationships discussed above are technologically feasible with the current state-of-the-art in software engineering, FOSS model development and distributed simulation, while some may need further research, yet others may not be considered realistic or efficacious given the current technology uptake. Irrespective, we believe that the combined application of multiple model reuse modes is a pertinent area of enquiry; an area which has received a shot in the arm with burgeoning use of FOSS and the rapidly advancing open science movement, and the accompanying increase of programming skills in the present generation of simulation modellers.

### 4.2. A hypothetical case study exemplifying the combined use of the three simulation reuse modes

The example is based on a published study by a co-author on modelling the UK National Blood Service (NBS) supply chain in the Southampton area.^84^ The objective of the modelling exercise was to investigate blood ordering policies across multiple hospitals served by a single NBS Processing, Testing, and Issuance (PTI) centre. This PTI centre was responsible for collecting donor blood, testing and processing it into products such as red blood cells (RBCs) and platelets, maintaining inventory and issuing blood units based on hospital demand.

Two models were developed using the commercial simulation package *Simul8*. The first was a conventional model that simulated the operations of one PTI and four hospitals as a single, integrated system. Since blood products have a limited shelf life and must be transfused before expiration, the model incorporated logic to decrement the shelf life of each unit (RBC or platelet) by one minute for every simulated minute. Given the high volume of blood units circulating in the system, this led to substantial computational demands and long experimentation times.

To reduce execution time in the original study, the authors implemented an approach aligned with *black-box model reuse*. Specifically, distributed simulation using HLA-RTI served as the black-box mechanism in the second implementation of the case study. This version comprised one Simul8 model for the PTI centre and four separate models for the individual hospitals, each reflecting a distinct demand profile. Message exchange between these distributed models, along with time synchronisation, was facilitated through a custom black-box HLA/RTI infrastructure.^84^

Thus, the existing study already exemplifies one mode of model reuse: black-box integration. For the purposes of the hypothetical scenario, the remaining part of this section will extend the case study to explore the two other modes of simulation model reuse: reuse enabled by open science tools and reuse through modelling languages and translators.

*Model reuse through open science tools*: At the time of the original study, Southampton had approximately 16 hospitals, all served by a single NBS PTI centre.^84^ Key processes such as physicians requesting blood based on planned clinical activities, checking local hospital inventory, placing orders with the PTI, performing cross-matching, assigning inventory (blood assigned to patients for a clinical procedure), transfusing blood units and expiration of inventory, were broadly similar across the four hospitals that were modelled. However, notable differences existed in terms of hospital characteristics, including size (e.g. large, medium, small), number of physicians, maximum inventory capacity, ordering strategies and inventory policies. Given that multiple hospitals had largely similar operational processes, model reuse using open-source tools such as SimPy could have significantly accelerated the development of individual hospital sub-models. For example, if the FOSS models were developed adhering to frameworks such as Sharing Tools and Artefacts for Reusable Simulations (STARS),^45^ it would enable the hospitals, which are part of the same UK NHS ecosystem, to preview the models, validate the conceptual model with user-centric requirements, and download and reuse the model code. This would substantially decrease the time needed to develop a FOSS model from scratch. This illustrates the relevance and value of reuse through open science tools.

*Reuse through modelling languages and translators*: The NHS delivers healthcare through a network of NHS Trusts. One category of these is Acute Trusts, which provide secondary care in hospitals and specialised medical facilities, including Emergency Departments (EDs). These Trusts typically have a high degree of autonomy in procurement and decision-making. Returning to our hypothetical example, let us assume that the 16 hospitals in Southampton are managed by three Acute NHS Trusts (Trusts A, B and C), all of which are interested in developing simulation models to represent the processes of their respective local blood banks, intending to link these models to the existing NBS PTI model. Based on factors such as available finances, technical expertise within the Trusts’ workforce and levels of confidentiality protocols in place, Trust A opts to invest in Simul8, whereas Trusts B and C choose to leverage FOSS by adopting SimPy, recognising the benefits of model reuse. In this example, the Simul8 model for Trust A is akin to a black-box since neither the data, the model logic, nor the model itself is shared. This is in contrast to white-box sharing and reuse of SimPy models by Trusts B and C. Interestingly, the mixing and matching of black versus white-box reuse helps accommodate the varying levels of confidentiality mentioned earlier.

For both the Simul8 and SimPy models to integrate with the NBS PTI model, appropriate interfaces for HLA-RTI are required to enable message exchange and simulation time synchronisation within the overarching simulation federation. Here, the third mode of model reuse, namely, reuse through modelling languages and transformations, becomes critical. This approach can automate the generation of the interface (or ‘glue’) code needed to facilitate interaction between both Simul8 and SimPy models and the HLA-RTI black-box. By doing so, it significantly reduces the time and complexity that would otherwise be involved in manually implementing such interoperability. This underscores the importance of our third mode of simulation model reuse, which centres on modelling languages and code transformations.

## 5. Conclusion

Model reuse has the potential to reduce development time in simulation modelling of healthcare and avoid reinventing the wheel in each new project. Reuse does not only have benefits in reducing development time but can also allow the user to benefit from validation carried out by the original developer and aid with experimentation. We believe that increased model reuse will help increase the day-to-day use of simulation to support decision-making for healthcare process improvements.

As discussed, while there is a well-established hierarchy of model reuse, from partial reuse to complete model replication, our work extends this framing by emphasising practical enablers. The triad of open science principles, modelling languages and black-box components method offers concrete means to support operational reuse in contemporary simulation practice. Distributed simulation provides a framework for black-box model reuse with trusted federate models being reused and applied within a new simulation project. The standards that have been developed within the distributed simulation community also make reuse easier. An important driver of model reuse is the accessibility of research, and open science principles have clear links to model reuse. Improving the availability and discoverability of models, as well as ensuring licensing is clear and documentation is available, comprehensive and easily understood will all improve the chances of a model being reused. A DSML, which is designed to enable reuse by encoding knowledge about the domain within the modelling language, can be a useful tool. DSMLs allow for grey-box reuse, reusing generic elements of a healthcare model and including elements that are specific to the particular project. Writing models in standard modelling language or DSML may also give them a longer life than writing them in off-the-shelf packages, which can become obsolete.

While these three modes of model reuse have seen significant research both within and outside the context of simulation development for healthcare in the past, the concrete mechanisms for synergies between the modes are less well understood. There are open research challenges around the development of concrete mechanisms and tools for leveraging the relationships between the three different modes, including for the potential offered by recent developments in artificial intelligence – for example, for generating computer code from natural-language descriptions^99,100^ or for managing arguments about simulation studies.^101^ Recent moves towards standardised data schemata and data exchange mechanisms (e.g. data dictionaries (https://www.datadictionary.nhs.uk/) or protocols like FHIR) will also be beneficial for simulation reuse by standardising the data-management pipelines required to connect simulations and real-world data. We invite the community to contribute to addressing these challenges.
